# *MEST* mediates the impact of prenatal bisphenol A exposure on long-term body weight development

**DOI:** 10.1186/s13148-018-0478-z

**Published:** 2018-04-20

**Authors:** Kristin M. Junge, Beate Leppert, Susanne Jahreis, Dirk K. Wissenbach, Ralph Feltens, Konrad Grützmann, Loreen Thürmann, Tobias Bauer, Naveed Ishaque, Matthias Schick, Melanie Bewerunge-Hudler, Stefan Röder, Mario Bauer, Angela Schulz, Michael Borte, Kathrin Landgraf, Antje Körner, Wieland Kiess, Martin von Bergen, Gabriele I. Stangl, Saskia Trump, Roland Eils, Tobias Polte, Irina Lehmann

**Affiliations:** 1 Department of Environmental Immunology, Helmholtz Centre for Environmental Research (UFZ), Leipzig, Germany; 2 Department of Dermatology, Venerology and Allergology, Leipzig University Medical Center, Leipzig, Germany; 3 Department Molecular Systems Biology, Helmholtz Centre for Environmental Research (UFZ), Leipzig, Germany; 4 Institute of Forensic Medicine, University Hospital Jena, Jena, Germany; 5 Core Unit for Molecular Tumor Diagnostics (CMTD), National Center for Tumor Diseases (NCT) Dresden, 01307 Dresden, Germany; 6 German Cancer Consortium (DKTK), Dresden, Germany; 7 German Cancer Research Center (DKFZ), 69120 Heidelberg, Germany; 8 German Cancer Research Center (DKFZ), Division of Theoretical Bioinformatics, Heidelberg, Germany; 9 German Cancer Research Center (DKFZ), Genomics and Proteomics Core Facility, Heidelberg, Germany; 10 Medical Faculty, Rudolf-Schönheimer-Institute of Biochemistry, University of Leipzig, Leipzig, Germany; 11 Children’s Hospital, Municipal Hospital “St. Georg”, Leipzig, Germany; 12 LIFE-Leipzig Research Centre for Civilization Diseases, University of Leipzig, Leipzig, Germany; 13 Hospital for Children and Adolescents-Centre for Pediatric Research, University of Leipzig, Leipzig, Germany; 14 Faculty of Biosciences, Pharmacy and Psychology, Institute of Biochemistry, University of Leipzig, Leipzig, Germany; 15 Institute of Agriculture and Nutritional Sciences, Martin Luther University Halle-Wittenberg, Halle (Saale), Germany; 16 Competence Cluster for Nutrition and Cardiovascular Health (nutriCARD), Halle-Jena Leipzig, Germany; 17 German Cancer Research Center (DKFZ), Heidelberg Center for Personalized Oncology, DKFZ-HIPO, Heidelberg, Germany; 18 Berlin Institute of Health and Charité-Universitätsmedizin Berlin, Center for Digital Health, Berlin, Germany; 19 Health Data Science Unit, Heidelberg University Hospital, Heidelberg, Germany; 20 Unit for Molecular Epidemiology, Berlin Institute of Health (BIH) and Charitè - Universitätsmedizin Berlin, Berlin, Germany

**Keywords:** EDC, Prenatal exposure, Infants, Obesity, LINA, Mice, Mesenchymal stem cells, Epigenetics, DNA methylation, Adipogenesis

## Abstract

**Background:**

Exposure to endocrine-disrupting chemicals can alter normal physiology and increase susceptibility to non-communicable diseases like obesity. Especially the prenatal and early postnatal period is highly vulnerable to adverse effects by environmental exposure, promoting developmental reprogramming by epigenetic alterations. To obtain a deeper insight into the role of prenatal bisphenol A (BPA) exposure in children’s overweight development, we combine epidemiological data with experimental models and BPA-dependent DNA methylation changes.

**Methods:**

BPA concentrations were measured in maternal urine samples of the LINA mother-child-study obtained during pregnancy (*n* = 552), and BPA-associated changes in cord blood DNA methylation were analyzed by Illumina Infinium HumanMethylation450 BeadChip arrays (*n* = 472). Methylation changes were verified by targeted MassARRAY analyses, assessed for their functional translation by qPCR and correlated with children’s body mass index (BMI) *z* scores at the age of 1 and 6 years. Further, female BALB/c mice were exposed to BPA from 1 week before mating until delivery, and weight development of their pups was monitored (*n* ≥ 8/group). Additionally, human adipose-derived mesenchymal stem cells were treated with BPA during the adipocyte differentiation period and assessed for exposure-related epigenetic, transcriptional and morphological changes (*n* = 4).

**Results:**

In prenatally BPA-exposed children two CpG sites with deviating cord blood DNA-methylation profiles were identified, among them a hypo-methylated CpG in the promoter of the obesity-associated mesoderm-specific transcript (*MEST)*. A mediator analysis suggested that prenatal BPA exposure was connected to cord blood *MEST* promoter methylation and *MEST* expression as well as BMI *z* scores in early infancy. This effect could be confirmed in mice in which prenatal BPA exposure altered *Mest* promoter methylation and transcription with a concomitant increase in the body weight of the juvenile offspring. An experimental model of in vitro differentiated human mesenchymal stem cells also revealed an epigenetically induced *MEST* expression and enhanced adipogenesis following BPA exposure.

**Conclusions:**

Our study provides evidence that *MEST* mediates the impact of prenatal BPA exposure on long-term body weight development in offspring by triggering adipocyte differentiation.

**Electronic supplementary material:**

The online version of this article (10.1186/s13148-018-0478-z) contains supplementary material, which is available to authorized users.

## Background

Exposure to endocrine-disrupting chemicals (EDCs) during critical windows in development can permanently alter normal physiology and increase susceptibility to diseases like obesity, asthma, or cancer later in life [1]. Especially the prenatal and early postnatal period is highly vulnerable to EDC exposure as it is the time of developmental programming important for organogenesis and tissue differentiation [2]. The growing knowledge about the human epigenome emphasized the importance of environmental exposure-related epigenetic modifications predisposing an individual to the development of disease. Understanding the underlying effects leading to a disruption in epigenetic programming by EDCs during fetal development is important and might aid future prevention strategies for such diseases.

One EDC with a well-described impact on the human epigenome during development is bisphenol A (BPA). BPA is a chemical used in the manufacturing of polycarbonate plastics and epoxy resins contained in a variety of consumer products. It is readily released to the environment leading to extensive human exposure in industrialized countries [3, 4]. BPA has been detected in human blood, urine, adipose tissue, breastmilk, and also in placental tissue and amniotic fluid [3], suggesting that exposure already starts during the sensitive prenatal phase. BPA is classified as an endocrine disruptor because of its ability to mimic hormone activity, for example, through estrogen-, and peroxisome proliferator-activated receptor gamma (PPARγ) signaling [5, 6]. After oral administration BPA is rapidly biotransformed to glucuronidated BPA in the liver via UDP-glucuronosyltransferase (UGT) and is eliminated by urinary excretion within 24 h [7, 8]. Complementary, studies in rats suggest that BPA metabolism might change during pregnancy due to alterations in UGT isoforms and expression level [9]. In addition, decreased UGT levels in fetal liver can lower the excretion capacity for BPA, making the fetus even more vulnerable to environmental pollutant EDC exposure [10–12]. So far, data on human BPA metabolism during pregnancy or early childhood are missing, but it seems reasonable to assume that also in pregnant women and in the developing fetus a lower excretion capacity might increase their vulnerability to BPA exposure with potential consequences for children’s later disease development.

In this context, BPA is highly discussed in terms of increasing the risk for obesity pathology but only few controversial studies on human prenatal BPA exposure exist so far [13–15]. Although animal studies are available to a greater extent, derived results are inconsistent and mechanistic investigations, for example, regarding underlying BPA-related epigenetic changes, are lacking. Epigenetic alterations related to BPA exposure have previously been associated with an increased risk of carcinogenesis [16–18] in rodent models of hepatic and prostate cancer. So far, no data on BPA-induced epigenetic modifications leading to overweight development exist.

Therefore, the aim of the present study was to analyze epigenetic alterations in the cord blood of prenatally exposed children and their potential link to overweight development as part of the German prospective LINA mother-child cohort. Findings from our epidemiological study were validated by applying an experimental mouse model for prenatal BPA exposure and an in vitro stem cell differentiation model demonstrating the impact of BPA exposure on adipocyte development.

## Methods

### LINA study design and sample collection

The LINA cohort study (Lifestyle and environmental factors and their Influence on Newborns Allergy risk) recruited 622 pregnant mothers (629 mother-child-pairs) between May 2006 and December 2008 in Leipzig, Germany, to investigate how environmental factors in the pre- and postnatal period influence disease risks later in children’s life [19–21]. Mothers suffering from immune or infectious diseases during pregnancy were excluded from the study.

Six hundred six mother-child-pairs participated in the year 1, 420 in the year 6 follow-up. Standardized questionnaires were administered during pregnancy (34th week of gestation) and annually thereafter, collecting general information about study participants, about housing and environmental conditions as well as about personal lifestyle. At the age of 1 and 6 years, height and body weight of the children were assessed during clinical visits. BMI *z* score were calculated according to the WHO references [22]. All questionnaires were self-administered by the parents. Participation in the study was voluntary and written informed consent was obtained from all participants. The study was approved by the Ethics Committee of the University of Leipzig (file ref. # 046–2006, #206–12-02072012).

### Analyses of urinary bisphenol A concentration in human samples

BPA quantification was carried out for 552 maternal urine samples (34th week of gestation) using a multianalyte procedure as described by Feltens et al. [23] and in more detail in the supplementary material. Absolute concentrations of BPA were calculated based on calibration curves and normalized to urinary creatinine concentrations as previously described [24].

### In vivo mouse model

BALB/c mice (6–8 weeks of age) were obtained from the Elevage Janvier Laboratory (Le Genest St Isle, France). Mice were bred and maintained in the animal facility at the University of Leipzig (Germany) and housed under conventional conditions with 23 °C room temperature, 60% humidity, and 12 h day/night rhythm. Cages were bedded with LIGNOCEL® bedding material (fine particles < 200 μm 0.2%). Mice received phytoestrogen-free diet (C1000 from Altromin, Lage, Germany) and water ad libitum from custom-built glass bottles to avoid contamination with BPA. All animal experiments involved groups of 4–6 mice/cage and were performed according to institutional and state guidelines. The Committee on Animal Welfare of Saxony approved animal protocols used in this study.

Dams were exposed to 5 μg/ml BPA (Sigma Aldrich, Munich, Germany) via the drinking water 1 week before mating until delivery of the offspring. For each exposure group (control or BPA), the exposure protocol was performed at least two times in at least three dams (each with 2–5 pups). Serum was collected from dams at the end of the BPA exposure. 1 week after delivery, pups were weighed two times per week and a mean weight per week was calculated for each mouse. At the end of the observation period (10 weeks), whole body composition (fat mass and lean mass) was determined in awake mice based on nuclear magnetic resonance technology using an EchoMRI700™ instrument (Echo Medical Systems, Houston, TX, USA) in the offspring of control and BPA exposed dams. Further, DNA-methylation analysis (MassARRAY) as well as gene expression analysis was performed in visceral fat tissue as described below in 10-week-old offspring. For measurement of fat mass/lean mass, MassARRAY and gene expression analysis, we used at least four mice per group from two to four dams (to avoid litter effects), but in any case with an equal number of male and female mice.

### Murine BPA ELISA

BPA concentration in serum was detected with BPA Assay Kit (Immuno-Biological Laboratories, Hamburg, Germany). Serum samples, enzyme-labeled BPA and anti-BPA serum were added to a pre-coated microtiter plate with anti-rabbit IgG and incubated for 1 h at room temperature. After washing, TMB was added as substrate and color reaction was detected at 450 nm. BPA serum concentration was calculated from a standard curve with a detection range from 0.3 to 100 ng/ml. Measured BPA serum levels in adult mice reached 19 ng/ml.

### In vitro adipocytes model

Human adipose-derived mesenchymal stem cells (MSC; ATCC®, PCS-500-011; #59753760) and culture media were purchased from LGC Standards (Wesel, Germany). For adipocyte differentiation MSCs at passage 1–3 were seeded at 9600 cells/cm^2^ and were cultured with Adipocyte Differentiation Initiation Medium (ADIM; ATCC Adipocyte Differentiation Toolkit PCS-500-050) for 4 days. Thereafter, Adipocyte Maintenance Medium (ADMM; ATCC® Adipocyte Differentiation Toolkit PCS-500-050) was applied for the subsequent 11 days. Media was changed every 2 to 4 days according to the manufacturer’s instructions. During the entire differentiation period, cells were exposed to 10 or 50 μM BPA (Sigma Aldrich, Munich, Germany) or a solvent control (0.05% ethanol); freshly added after every medium change. The differentiation process was monitored in real-time by the impedance-based xCELLigence SP System (ACEA Biosciences Inc., San Diego, USA) on a microelectrode 96 well E-View-Plate (ACEA Biosciences Inc.). The growth rate was monitored every 10 min by electrical impedance measurements that were paused for media changes and a Cell Index was calculated, by normalization to a blank value for each well. After differentiation, cells were stained with Oil Red O for 45 min for triglyceride depots and mRNA was extracted (see supplementary material).

A MTT assay (3-(4,5-Dimethylthiazol-2-yl)-2,5-diphenyltetrazoliumbromid) was applied to a BPA concentration series to identify appropriate non-toxic concentrations for the in vitro assay. For details, see supplementary material.

### DNA methylation analysis via 450 K array

Genomic DNA was isolated from cord blood samples using the QIAmp DNA Blood Mini Kit (Qiagen, Hilden, Germany) followed by bisulfite conversion using the EZ-96 DNA Methylation Kit (Zymo Research Corporation, Orange, USA) according to the manufacturer’s recommendations. All samples subsequently subjected to DNA methylation analyses passed the initial quality control check (*n* = 472).

A genome-wide DNA methylation screen was performed based on the Infinium HumanMethylation450 BeadChip (Illumina, San Diego, USA) array (see supplementary material). Data were normalized using the SWAN (subset-quantile within array normalization) method of the minfi R package [25]. DNA methylation values, described as beta values (β), were recorded for each locus in each sample. For statistical analyses β values were logit transformed to *M* values [26].

To account for potential differences in cell composition, we used publically available FACS data of sorted cord blood cells [27] implemented in the R package *FlowSorted.CordBloodNorway.450 k: Illumina HumanMethylation data on sorted cord blood cell populations* (version 1.4.0) [28] and the estimateCellCounts-function of the minfi R package. The resulting information on CD4+ T cells, CD8+ T cells, natural killer cells, B cells, granulocytes, and monocyte proportions were used as confounders in the subsequent regression analysis. In addition, previously identified factors with an impact on cord blood methylation were considered as confounders including the maternal vitamin D level [29], prenatal benzene exposure, maternal smoking [30, 31], and maternal stress during pregnancy [32]. Differentially methylated CpGs were determined by applying logistic regression models on methylation *M* values [33] adjusted for the confounders mentioned above. A Bonferroni correction was applied on obtained *p* values resulting in a significance level of *p* < 2.37E-7. For details, see supplementary material.

### MassARRAY validation of DNA methylation

**Fig. 1:**
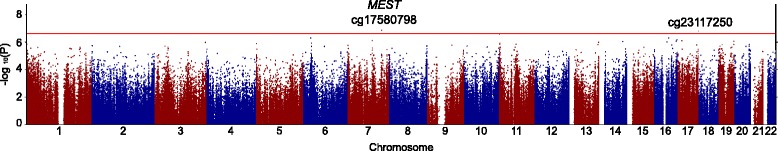
Epigenome wide analysis and *MEST* methylation assessment. Manhattan-Plot from 450 K array comparing children prenatally exposed to high vs. low BPA. Shown are significant CpGs observed in cord blood that passed threshold for Bonferroni correction (red threshold line, *p* < 2.37E-7)

A quantitative DNA methylation analysis of the human *MEST* promoter was performed in cord blood samples of the LINA cohort and in in vitro adipocytes using Sequenom’s MassARRAY platform as described previously [31]. Briefly, a PCR amplicon was designed on the reverse strand covering chr7: 130,132,068-130,132,287 including cg17580798 (Fig. 1, *MEST* forward primer: aggaagagagTTTAGAGGTAGTTTTAGTTYGG*,* reverse primer: cagtaatacgactcactatagggagaaggctCCRCTACTAACCAACTCTAC with an annealing temperature of 52 °C). A total of 24 CpGs was covered by the amplicon. For analysis, all high mass, duplicate, and silent peaks were excluded from the analysis retaining 14 CpGs, which were averaged and used for further analysis.

gDNA extracted from murine adipose tissue F1 (*n* ≥ 3, per treatment group) was bisulfite converted using the EZ DNA Methylation kit (Zymo Research, Freiburg, Germany) and subjected to MassARRAY analysis. Genome coordinates of the human *MEST* promoter were lifted over to the mouse genome assembly mm10 and corresponding primer pairs on the forward strand were designed (*Mest* forward primer: aggaagagagAGGAGGTTTGTGTTTTTAATG, reverse: cagtaatacgactcactatagggagaaggctCACCCACTTCTTTTCTACC, annealing temperature: 60 °C, amplicon coordinates: chr6: 30,737,347-30,737,692).

### Gene expression analysis

Gene expression analysis in samples of the LINA cohort was performed as described earlier [31] and in more detail in the supplementary material. Briefly, intron-spanning primers were designed, and UPL probes were selected by the Universal Probe Library Assay Design Center. After a preamplification step qPCRs were conducted on 96.96 Dynamic Array (Fluidigm, San Francisco, CA, USA). Gene expression values were determined with *glycerinaldehyd-3-phosphat-dehydrogenase (GAPD)* and *glucuronidase beta (GUSB)* as reference genes and normalized to the lowest measured value. The following primer pairs were used for *MEST* (primer-for 5′- atcgtggaagcgcttttg, -rev 5′- gaccagatcgattctgcttgta, UPL50) and the reference genes *GAPD* and *GUSB* (primer-for 5′-gctctctgctcctcctgttc, -rev 5′-acgaccaaatccgttgactc, UPL 60; -for 5′-cgccctgcctatctgtattc, -rev 5′-tccccacagggagtgtgtag, UPL 57, respectively).

*Mest* expression in murine fat tissue was assessed by qPCR. Expression values were determined by applying the 2-ΔΔCT method and normalized to *Gapdh and UBC (−for 5′-*gtctgctgtgtgaggactgc*, rev 5′-* cctccagggtgatggtctta *UPL 77).*

Furthermore, gene expression of *peroxisome proliferator-activated receptor gamma* (*PPARG)*, *sterol regulatory element-binding factor 1 (SREBF1)*, *lipoprotein lipase (LPL)*, *leptin (LEP)*, *fatty acid synthase (FASN)*, *mesoderm specific transcript (MEST*), *estrogen receptor alpha* (*ESR1)*, and *insulin receptor substrate 2* (*IRS2)* was assessed by qPCR of human in vitro derived adipocytes.

All used primer pairs are listed in Additional file 1: Table S1. All primers were designed as intron spanning assays to assure specificity.

### Statistical analyses

To test the equal distribution of parameters in the analyzed sub-cohort and the entire LINA cohort, the chi-squared test was performed. LINA study data were evaluated by STATISTICA for Windows, Version 12 (Statsoft Inc., USA). 450 k data were analyzed and processed using the R packages minfi and qqman (R version 3.3.1, R Foundation for Statistical Computing).

BPA concentrations and DNA methylation levels were log transformed for further statistical analyses. To assess longitudinal associations of gene expression and weight development, a generalized estimating equation (GEE) model was applied. Mediator models for the connection of prenatal BPA exposure with the methylation status and children’s BMI *z* scores were analyzed using the PROCESS macro v2.16.3 in IBM SPSS Statistics version 22 (IBM Corps., USA) [34]. All models were adjusted for the gender of the child, smoking during pregnancy, parental school education, solid food introduction, gestational week at delivery, number of household members, and early delivery (< 37 weeks of gestation). Weight-related confounders were chosen according to a literature review.

Experimental data sets from murine and in vitro studies were processed and analyzed in GraphPad PRISM 7.02 for windows (GraphPad Software, Inc.). All *p* values ≤ 0.05 were considered to be significant.

## Results

### General study characteristics

Our analyzed sub-cohort was comprised of the 408 children for whom data on prenatal BPA exposure and the cord blood methylation status were available. General characteristics of the study participants (gender, birth weight, gestational week at delivery, smoking during pregnancy, parental school education, household members, breastfeeding, and introduction to solid food) of the sub-cohort (*n* = 408) were not different from the total LINA cohort (*n* = 629) as shown in Table 1. Median urinary BPA concentrations at pregnancy were 12.7 ng/mg creatinine. Low BPA exposure was defined as < 7.6 ng/mg creatinine (< 25%; 1st or lower quartile) and high BPA exposure as > 15.9 ng/mg creatinine (> 75%, 4th or upper quartile). BMI z scores at year 1 ranged from − 3.5 to 2.9 with a median of − 0.2, BMI *z* scores at year 6 ranged from − 2.2 to 4.2 with a median of 0.0.

**Table 1:** General study population characteristics

	Entire LINA cohort *n* (%), *n* = 629^a^	Analyzed sub-cohort *n* (%), *n* = 408	*χ* ^*2*^ *test* ^b^
Gender of the child			0.966
Female	302(48.0)	197(48.3)	
Male	327(52.0)	211(51.7)	
Birth weight			0.941
≤ 3000 g	123(19.6)	68(16.7)	
> 3000–3500 g	242(38.5)	157(38.5)	
> 3500–4000 g	192(30.6)	129(31.6)	
> 4000 g	71(11.3)	54(13.2)	
Gestational week at delivery			0.834
< 37 weeks	25(4.0)	10(2.5)	
37–40 weeks	389(62.0)	255(62.5)	
> 40 weeks	214(34.0)	143(35.0)	
Smoking during pregnancy			0.833
Never	534(84.9)	358(87.7)	
Occasionally	47(7.4)	23(5.6)	
Daily	48(7.6)	27(6.6)	
Parental school education^**c**^			0.969
Low	16(2.5)	8(2.0)	
Intermediate	144(22.9)	96(23.5)	
High	469(74.6)	304(74.5)	
Household members			0.932
2	33(5.2)	20(4.9)	
3	365(58.0)	257(63.0)	
> 4	203(32.3)	129(31.6)	
Breastfeeding exclusive			0.968
1–3 months	112(17.8)	69(16.9)	
1–6 months	190(30.2)	121(29.7)	
1–12 months	254(40.4)	172(42.2)	
Introduction to solid food			0.897
1–3 months	23(3.7)	11(2.7)	
4–6 months	251(39.9)	156(38.2)	
7–12 months	305(48.5)	205(50.2)	
Urinary BPA concentration during pregnancy			0.263^d^
Median [ng/mg creatinine]	12.7	12.7	
IQR^e^ [ng/mg creatinine]	7.5–16.0	7.6–15.9	
BMI *z* score at year 1	*n* = 564	*n* = 366	
Median	− 0.24	− 0.16	
IQR	− 0.90–0.35	− 0.79-0.43	
BMI *z* score at year 6	*n* = 303	*n* = 192	
Median	− 0.02	0.05	
IQR	− 0.67-0.50	− 0.51-0.54	

^a^*n* may be different from 629 due to missing data

^b^Calculated using the chi-squared test for cross relationship

^c^Low = 8 years of schooling (‘Hauptschulabschluss`); intermediate = 10 years of schooling (`Mittlere Reife`); high = 12 years of schooling or more (`(Fach-)hochschulreife’)

^d^*p*-value derived by Student’s *t* test between group means

^e^IQR: inter quartile range (25th to 75th percentile)

#### Prenatal BPA exposure and cord blood DNA methylation of MEST

As there is growing evidence that epigenetic mechanisms such as DNA methylation changes can contribute to prenatal programming of diseases, the potential impact of BPA on children’s DNA-methylation pattern was assessed. Using bisulfite converted gDNA from cord blood, genome-wide changes in DNA methylation were evaluated by applying Illumina Infinium HumanMethylation450 BeadChip arrays. Differentially methylated CpG sites were computed using a regression model for high (fourth quartile) versus low (first quartile) prenatal BPA exposure. Two CpGs passed the threshold for Bonferroni correction (Fig. 1a and Table 2), including a hypo-methylated CpG (cg17580798) in the *MEST* promoter (chr7:130132199, *p* = 1.35E-07) and cg23117250 (chr17: 80649886, intronic, *p* = 1.55E-07) that is located in an intron of *RAB40B*. *RAB40B* encodes for a poorly characterized protein proposed to be involved in vesicle transport [35] and cancer progression [36].

**Table 2:** Epigenome-wide association study (EWAS) comparing children prenatally exposed to high vs. low BPA. Shown are significant CpGs observed in cord blood that passed Bonferroni correction

CpG	Chromosome	Position	Region	Host gene	*p* value ^a^	Δ β^b^
cg17580798	7	130,132,199	Promoter	*MEST*	1.35E-07	−1.8%
cg23117250	17	80,649,886	Intron	*RAB40B*	1.55E-07	−2.0%

^a^*p* values are derived from a regression model with prenatal vitamin D level, prenatal benzene exposure, maternal smoking, maternal stress, and cord blood cell composition as confounding parameters

^b^Methylation differences are shown as Δ methylation values (β)

Thus, we focused further analyses on cg17580798 since *MEST*, as a member of the alpha/beta hydrolase superfamily, is reported to control the initial phase of early adipose tissue expansion by regulating adipocyte size [37]. Although cg17580798 is located in the first intron of *MEST*, ENCODE histone modification data suggest that it is a promoter region. That indeed this region is potentially transcriptionally regulating is supported by its overlap with a DNase I hypersensitivity cluster.

*MEST* promoter methylation around cg17580798 was validated by MassARRAY (see Additional file 2: Figure S1). The MassARRAY amplicon included 24 CpG sites of which 14 CpG sites passed quality control and were averaged as “total promoter methylation.” BPA exposure was associated with total promoter methylation (adj.MR: 0.88, 95% CI (0.80, 0.97), *p* = 0.010), as well as methylation of the CpG corresponding to cg17580798 only (adj.MR: 0.90, 95% CI (0.82, 0.99), *p* = 0.033). The methylation difference between low and high BPA exposure was 2.6 and 2.3%, respectively.

#### BPA associates with MEST promoter methylation and MEST expression in cord blood

**Fig. 2:**
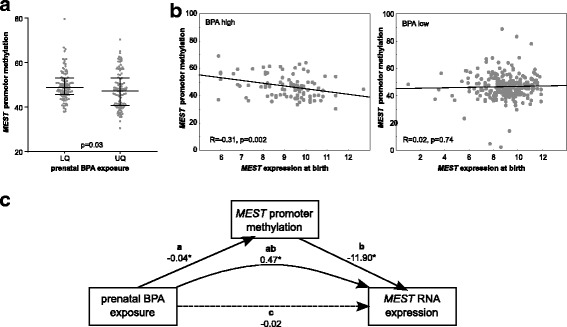
Association of BPA with *MEST* promoter methylation and *MEST* expression in cord blood **a** *MEST* promoter methylation (=mean of MassARRAY amplicon) in cord blood of low (< 25%; lower quartile (LQ), *n* = 102) and high (> 75%, upper quartile (UQ), *n* = 101) BPA-exposed children. *p* value from MWU-test. **b** *MEST* promoter methylation and expression in cord blood are correlated in children with high prenatal exposure to BPA (UQ, *n* = 94), while *MEST* expression is not correlated with *MEST* promoter methylation in lowly exposed children (remaining 75%, *n* = 267). *R* and *p* values from Spearman correlation. **c** Mediator model for the association of prenatal BPA exposure, cord blood *MEST* promoter methylation and expression. Models were adjusted for gender of the child, smoking during pregnancy, parental school education, solid food introduction, week of gestation at delivery, number of household members, and early delivery. Shown are effect sizes with *p < 0.05

*MEST* expression was measured in 408 cord blood samples of the LINA cohort. Complete information of *MEST* methylation status, *MEST* expression and prenatal BPA exposure was available for 361 children. High prenatal BPA exposure (> 75%, upper quartile (UQ)) was associated with a decrease in *MEST* promoter methylation at birth as determined by MassARRAY (Fig. 2a). Further, this *MEST* promoter hypomethylation was associated with an increase in *MEST* RNA expression, which was not observed in lowly exposed children (Fig. 2b). There was no direct effect of prenatal BPA exposure on cord blood *MEST* expression. However, applying a mediation model using PROCESS in SPSS, prenatal BPA exposure was linked indirectly to *MEST* expression by *MEST* promoter methylation (ab = 0.47, 95% CI (0.07, 1.24); Fig. 2c and Additional file 1: Table S2) at time of birth. Furthermore, *MEST* expression at birth was positively correlated with BMI *z* scores (adj.MR: 1.13, 95% CI (1.02, 1.26), *p* = 0.024).

#### BPA increases risk for childhood overweight development via MEST methylation

**Fig. 3:**
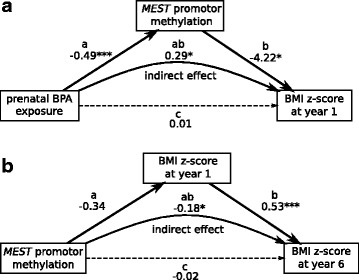
Mediator models **a** for the association of prenatal BPA exposure with *MEST* promoter methylation and children’s BMI *z* scores at year 1, **b** for the association of cord blood *MEST* methylation with children’s BMI *z* score at years 1 and 6. Models were adjusted for gender of the child, smoking during pregnancy, parental school education, solid food introduction, week of gestation at delivery, number of household members, and early delivery. Shown are effect sizes with *p < 0.05; ***p < 0.001

In addition, we were interested whether the changes in *MEST* promoter methylation that were associated with BPA exposure have relevance for the later weight development of the child. Therefore, we applied a mediator analysis, adjusted for weight-related confounders, to assess the impact of prenatal BPA exposure on children’s BMI *z* scores at year 1, which might be mediated by neonatal *MEST* promoter methylation. Indeed the mediation analysis indicates that the effect of prenatal BPA exposure on BMI *z* scores is mediated by *MEST* promoter methylation in cord blood (ab = 0.29, 95% CI (0.03, 1.09), Fig. 3a and Additional file 1: Table S3). Furthermore, the impact of cord blood *MEST* promoter methylation on BMI *z* scores at year 6 was mediated by the BMI *z* scores at year 1 (ab = − 0.18, 95% CI (− 0.51, − 0.06), Fig. 3b and Additional file 1: Table S4).

#### MEST expression is associated with longitudinal weight development

The longitudinal impact of altered *MEST* expression at birth due to prenatal BPA exposure was calculated using a generalized estimating equation (GEE) model including BMI *z* scores and *MEST* expression at birth and year 6 as well as weight related confounders. We found that a longitudinally higher *MEST* expression at birth and year 6 was positively correlated with longitudinal weight development at birth and year 6 (adj.RR: 1.03, 95% CI (1.00, 1.07), *p* = 0.021).

#### In vivo mouse model: impact of prenatal BPA exposure on weight development

**Fig. 4:**
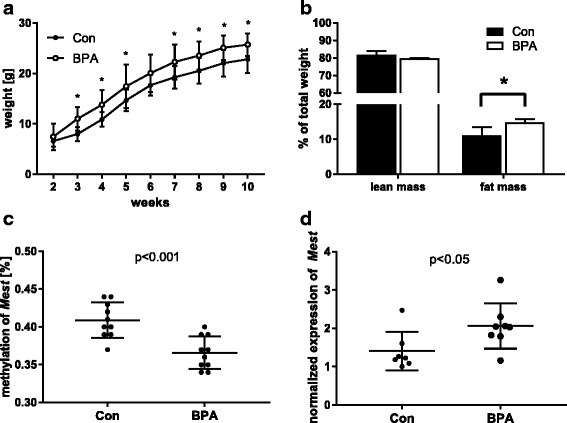
BPA effects in a murine in vivo model. **a** Impact of prenatal BPA exposure on weight development in the offspring. Shown are means and standard deviations from *n* ≥ 8/group and *p* values are derived from ANOVA. **b** Differentiation of offspring weight at 10 weeks in lean and fat mass. **c** Targeted MassARRAY *Mest* methylation analysis in fat tissue of 10-week-old offspring after prenatal BPA exposure compared to controls. **d** *Mest* expression analysis in visceral fat tissue of 10-week-old offspring after prenatal BPA exposure compared to controls. For **b**, **c**, and **d** data of *n* ≥ 4, mice is presented with *p < 0.05 and ***p < 0.001

To validate our findings from the LINA cohort and add further information on *Mest* methylation and expression in fat tissue, we applied a mouse model under standardized conditions. Mice offspring of BPA-exposed mothers were followed up until 10 weeks after delivery. Weight was assessed twice a week, beginning 1 week after delivery, and compared to unexposed control animals (Fig. 4a). Prenatally BPA exposed mice had a significantly higher weight over the entire observation period compared to unexposed control mice (*p* = 0.004, *p* value derived by ANOVA) with a mean difference of 0.85 g (week 2) to 3.04 g (weeks 3, 8, and 9). There was no gender difference in BPA-dependent weight development (Additional file 3: Figure S2). Furthermore, lean mass and fat mass were assessed at 10 weeks, with BPA-exposed mice showing a 53% higher fat mass than control mice (*p* = 0.013, Fig. 4b). *Mest* methylation and expression was assessed at 10 weeks in fat tissue samples. *Mest* methylation was reduced by 7% in BPA exposed mice (*p* < 0.001, Fig. 4c) with a corresponding increase in *Mest* expression by 2.1-fold in BPA exposed mice (*p* = 0.022, Fig. 4d).

#### In vitro model: impact of BPA exposure on adipocyte differentiation

**Fig. 5:**
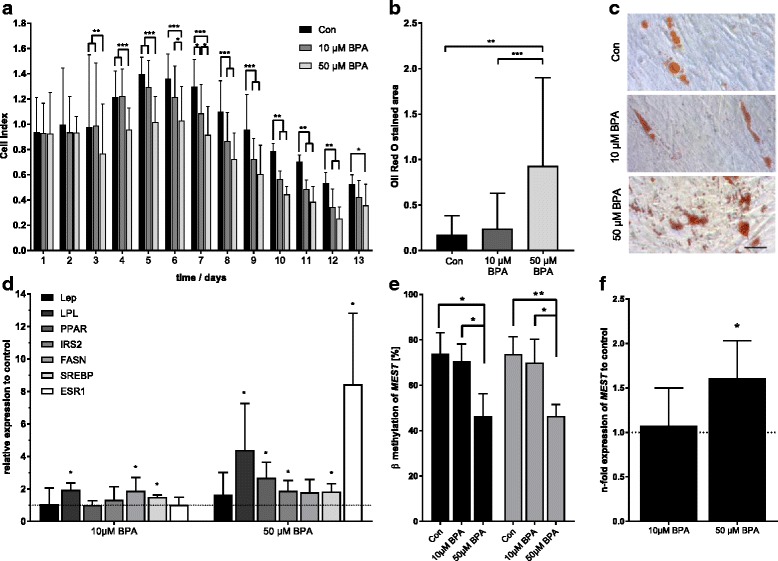
In vitro adipocyte differentiation from human MSCs: exposure to BPA (10 μM, 50 μM) compared to solvent control (EtOH 0.05%). **a** Real-time monitoring of cell differentiation (xCELLigence: normalized cell index) over a 17-day period (mean ± SD, *n* = 4). **b** Quantification of Oil Red O stained area (mean ± SD, *n* ≥ 20 from one experiment). **c** Exemplary histological Oil Red O staining of adipocytes (black bar = 100 μm). **d** qPCR data of genes involved in adipogenesis (*n* ≥ 3) normalized to EtOH control (*Lep* = leptin, *LPL* = lipoprotein lipase, *PPAR*γ = peroxisome proliferator activated receptor gamma, *IRS2* = insulin receptor substrate 2, *FASN* = fatty acid synthase, *SREBF*1 = sterol receptor element binding factor 1, *ESR1* = estrogen receptor alpha). **e** Targeted MassARRAY analysis of *MEST* promoter methylation, shown are the measurement of the single CpG cg17580798 covered by the amplicon (gray bars, *n* = 3) and the mean of the MassARRAY amplicon (black bars). **f** qPCR data of *MEST* (*n* ≥ 3, relative to EtOH control); *p < 0.05, **p < 0.01, ***p < 0.001 from Student’s *t* test/ANOVA

Differentiation of human MSC to adipocytes was monitored in real time using the impedance-based xCELLigence System. 10 or 50 μM BPA were applied during the entire differentiation period and compared to a solvent control (EtOH, 0.05%). BPA caused a dose-dependent decrease in cell index values after the differentiation initiation period compared to unexposed controls (Fig. 5a). Significance was reached from day 3 on for 50 μM and from day 5 on for 10 μM BPA until the end of the observational period. Oil Red O staining of lipid droplets showed significantly more droplets for 50 μM BPA (*p* < 0.001; Fig. 5b, c) but not for 10 μM BPA compared to unexposed control cells.

mRNA analysis of adipocyte-specific genes after 17 days of differentiation in the presence of 50 μM BPA revealed a significant upregulation of *PPAR*γ (2.2 ± 1.15-fold, *p* = 0.005), its target gene *LPL* (4.4 ± 2.6-fold, *p* = 0.029); *SREBF1* (1.8 ± 0.4-fold, *p* = 0.005), its target gene *IRS2* (1.9 ± 0.6-fold, *p* = 0.015; Fig. 2b), and *ESR1* (8.4 ± 3.8-fold, *p* = 0.006). For 10 μM BPA, a significant increase in gene expression was detected for *LPL* (1.9 ± 0.3-fold, *p* = 0.002), *SREBF1* (1.5 ± 0.1-fold, *p* < 0.001) and *FASN* (1.9 ± 0.7-fold, *p* = 0.046, Fig. 5d).

*MEST* methylation and expression was measured in differentiated adipocytes as shown in Fig. 5e, f. *MEST* promoter methylation (total and cg17580798) was decreased by 28% after exposure to 50 μM BPA compared to the control. In accordance, *MEST* expression was significantly increased in adipocytes exposed to 50 μM BPA (1.6 ± 0.4-fold, *p* = 0.027, Fig. 5e, f).

Our in vitro results are not influenced by any cytotoxic effects of BPA, as can be seen from the performed MTT assay (Additional file 4: Figure S3). There was no change in cell viability up to 50 μM BPA, although cells exposed to 100 μM BPA showed a slight but significantly lower cell viability after 48 h (*p* = 0.027).

## Discussion

Our study provides first evidence that prenatal BPA exposure causes epigenetic changes in the *MEST* promoter potentially contributing to overweight development in children with longitudinal effects until the age of 6 years (Additional file 5: Figure S4). Results from our experimental models support these epidemiological findings: prenatally BPA exposed mice showed hypo-methylation of the *MEST* promoter region and developed a significantly higher body weight compared to controls. Furthermore, a stimulating impact of BPA on adipocyte differentiation from human MSC was observed. Although these experimental data have to be interpreted with caution, since the applied exposure concentrations were quite different compared to the real human exposure situation, there is some evidence for an involvement of *MEST* in BPA-induced adipogenesis.

Results from this study may provide a first mechanistic explanation how prenatal BPA potentially exposure contributes to overweight development in the children. We identified two differentially methylated CpG sites in cord blood in association to prenatal BPA exposure, among them one hypo-methylated CpG in the *MEST* promoter. *MEST* is a paternally imprinted gene that encodes a member of the α/β hydrolase fold family, and its expression has been described to be associated with obesity [38–40], adipocyte size [37], and preadipocyte proliferation [41] in mouse and human studies. *MEST* knock-out mice showed reduced body weight and less obesity. *Mest* expression has been associated with variable obesity in mice and is attenuated by a positive energy balance [42]. High *Mest* expression was found in high-gainers even at only 1 week of high fat diet and may therefore be able to foreshadow food metabolism capacity in mice [43–45].

Recently, a link between prenatal BPA exposure and an epigenetic modification in the imprinted *Mest* gene was observed in a murine study. Trapphoff et al. reported hypo-methylation of the *Mest* promoter due to BPA exposure in murine oocytes [46]. Perinatal BPA exposure interferes with DNA methyltransferase 3a/3b (DNMT3A/DNMT3B) expression in mice, specifically affecting the de novo methylation of imprinted genes [47], which might be a contributing factor to the observed hypo-methylation. In this study, we show for the first time that also in humans prenatal BPA exposure is related to DNA methylation changes in the *MEST* promoter. It was already suggested that *MEST* methylation levels are associated with obesity risk in humans [48, 49]. Thus, our observed hypo-methylation in the *MEST* promoter may link prenatal BPA exposure to the overweight development in the offspring. In line with this hypothesis, we showed that *MEST* expression was associated with BMI increase on a longitudinal scale.

Although the observed methylation difference in the *MEST* promoter between BPA high and low exposed children in cord blood samples was only 1%, we nevertheless believe that this very small difference in the methylation status could be of biological relevance. *MEST* is expressed in mesenchymal tissue and also in MSCs, which are the source of adipose tissue, but not in blood cells. Since cord blood contains a sizeable number of MSCs, we suppose that the observed BPA-related hypo-methylation in the cord blood samples of our study relates to an expansion in the cord blood MSC fraction and *MEST* activation. Unfortunately, we were not able to test this hypothesis within our LINA study due to limited cell availability. However, data from an earlier study may support the idea that changes in specific cell populations in response to environmental exposure might be the cause of small DNA methylation differences observed in whole blood samples and, moreover, might be also of biological relevance if this particular cell population is involved in pathophysiology [30].

Since we were not able to isolate and test MSCs from our study participants, we applied an in vitro model to analyze the impact of BPA on MSCs. In adipocytes differentiated from BPA-exposed human MSCs, we showed a hypo-methylation of the *MEST* promoter region and an enhanced *MEST* expression. Although the applied BPA concentrations in this experimental model were much higher compared to the real exposure situation in humans, these data nevertheless may support the hypothesis that BPA induces *MEST* activation in human MSCs, which further corroborates a role of *MEST* in BPA-induced adipogenesis.

A limitation of this study is the missing information about maternal weight before and during pregnancy as a potential confounding factor. Further, BPA concentrations were measured in spot urine samples. BPA concentrations vary widely throughout the day and spot urine BPA concentrations only reflect exposure of the last 4–6 h [7]. Moreover, we cannot exclude the possibility of BPA contaminations introduced by tubing or reaction tubes during the storage and analytical procedure as pointed out to be critical by recent publications [50, 51]. However, samples were all stored in the same tubes and were analyzed at the same time, suggesting rather a systematic overestimation of the BPA concentration than a random contamination effect. The strength of our study is the combination of epidemiological data with in vivo and in vitro experimental models. For the first time, we performed a genome-wide DNA-methylation analysis in the cord blood of prenatally BPA-exposed children and found an epigenetic link between BPA exposure and overweight development.

## Conclusion

In conclusion, our study demonstrates that prenatal BPA exposure seems to be a contributing factor in the development of an early overweight phenotype by implicating epigenetic changes in the obesity-related gene *MEST*.

## Additional files

Additional file 1: **Table S1.** Primer for gene expression analysis. **Table S2.** Mediator model for the association of prenatal BPA exposure with cord blood *MEST* DNA methylation and expression (according to Fig. 1c). **Table S3.** Mediator model for the association of prenatal BPA exposure with cord blood *MEST* DNA methylation and children’s BMI *z* scores at year 1 (according to Fig. 3a). **Table S4.** Mediator model for the association of cord blood *MEST* DNA methylation and children’s BMI *z* scores at years 1 and 6 (according to Fig. 3b). (DOCX 45 kb)

Additional file 2: **Figure S1.** Shown are the location of the *MEST* gene on chromosome 7 (upper part), the 450 K array CpG in the *MEST* promoter (middle part) and the region covered by the MassARRAY amplicon within the promoter region (CpG sites are depicted in red). (PDF 32 kb)

Additional file 3: **Figure S2.** BPA effect on weight development assessed in a murine in vivo model stratified for gender. Shown are means and standard deviation from *n* ≥ 8 mice/group for all, female and male mice separately. *p* values are derived from ANOVA. (TIFF 1362 kb)

Additional file 4: **Figure S3.** MTT assay: MTT test for cell viability after exposure to BPA and the solvent control EtOH (0.05%), normalized to unexposed control, Student’s *t* test *p < 0.05, mean ± SD, *n* = 3. (JPEG 105 kb)

Additional file 5: **Figure S4.** Summary scheme: results overview and hypothesis indicating the influence of prenatal BPA exposure on *MEST* methylation and expression that is associated with adipocyte differentiation and overweight development in infant offspring. (PNG 19 kb)

## Abbreviations

95% CI: 95% confidence interval
BMI: Body mass index
BPA: Bisphenol A
Con: Control
DNMT: DNA methyltransferase
EDC: Endocrine-disrupting chemicals
*ESR1*: *Estrogen receptor alpha*
EtOH: Ethanol
*FASN*: *Fatty acid synthase*
*GAPDH*: *Glycerinaldehyd-3-phosphat-dehydrogenase*
gDNA: Genomic DNA
GEE: Generalized estimating equation
*GUSB*: *Glucuronidase beta*
*IRS2*: *Insulin receptor substrate 2*
*LEP*: *Leptin*
*LPL*: *Lipoprotein lipase*
*MEST*: *Mesoderm specific transcript*
MR: Mean ratio
MSC: Mesenchymal stem cells
MWU: Mann-Whitney *U* test
*PPARγ*: *Peroxisome proliferator-activated receptor gamma*
RR: Risk ratio
*SREBF1*: *Sterol regulatory element-binding factor 1*
UGT: UDP-glucuronosyltransferase
WHO: World Health Organization

## References

[1] Prusinski L, Al-Hendy A, Yang Q (2016). Developmental exposure to endocrine disrupting chemicals alters the epigenome: identification of reprogrammed targets. Gynecol Obstet Res.

[2] Gore AC, Heindel JJ, Zoeller RT (2006). Endocrine disruption for endocrinologists (and others). Endocrinology.

[3] Vandenberg LN, Hauser R, Marcus M, Olea N, Welshons WV (2007). Human exposure to bisphenol A (BPA). Reprod Toxicol.

[4] Calafat AM, Ye XY, Wong LY, Reidy JA, Needham LL (2008). Exposure of the US population to bisphenol a and 4-tertiary-octylphenol: 2003-2004. Environ Health Perspect.

[5] Janesick A, Blumberg B (2012). Obesogens, stem cells and the developmental programming of obesity. Int J Androl.

[6] Ross MG, Desai M (2013). Developmental programming of offspring obesity, adipogenesis, and appetite. Clin Obstet Gynecol.

[7] Volkel W, Colnot T, Csanady GA, Filser JG, Dekant W (2002). Metabolism and kinetics of bisphenol a in humans at low doses following oral administration. Chem Res Toxicol.

[8] Oppeneer SJ, Robien K (2015). Bisphenol a exposure and associations with obesity among adults: a critical review. Public Health Nutr.

[9] Inoue H, Tsuruta A, Kudo S, Ishii T, Fukushima Y, Iwano H, Yokota H, Kato S (2005). Bisphenol a glucuronidation and excretion in liver of pregnant and nonpregnant female rats. Drug Metab Dispos.

[10] Strassburg CP, Strassburg A, Kneip S, Barut A, Tukey RH, Rodeck B, Manns MP (2002). Developmental aspects of human hepatic drug glucuronidation in young children and adults. Gut.

[11] Burchell B, Coughtrie M, Jackson M, Harding D, Fournelgigleux S, Leakey J, Hume R (1989). Development of human-liver Udp-glucuronosyltransferases. Dev Pharmacol Ther.

[12] Pacifici GM, Franchi M, Giuliani L, Rane A (1989). Development of the glucuronyltransferase and sulphotransferase towards 2-naphthol in human fetus. Dev Pharmacol Ther.

[13] Valvi D, Casas M, Mendez M, Ballesteros-Gomez A, Luque N, Rubio S, Sunyer J, Vrijheid M (2013). Prenatal bisphenol a urine concentrations and early rapid growth and overweight risk in the offspring. Epidemiology.

[14] Braun JM, Lanphear BP, Calafat AM, Deria S, Khoury J, Howe CJ, Venners SA (2014). Early-life bisphenol a exposure and child body mass index: a prospective cohort study. Environ Health Perspect.

[15] Harley KG, Aguilar Schall R, Chevrier J, Tyler K, Aguirre H, Bradman A, Holland N, Lustig R, Calafat AM, Eskenazi B (2013). Prenatal and postnatal bisphenol a exposure and body mass index in childhood in the CHAMACOS cohort. Environ Health Perspect.

[16] Weinhouse C, Sartor MA, Faulk C, Anderson OS, Sant KE, Harris C, Dolinoy DC (2016). Epigenome-wide DNA methylation analysis implicates neuronal and inflammatory signaling pathways in adult murine hepatic tumorigenesis following perinatal exposure to bisphenol a. Environ Mol Mutagen.

[17] Cheong A, Zhang X, Cheung YY, Tang WY, Chen J, Ye SH, Medvedovic M, Leung YK, Prins GS, Ho SM (2016). DNA methylome changes by estradiol benzoate and bisphenol a links early-life environmental exposures to prostate cancer risk. Epigenetics.

[18] Faulk C, Kim JH, Anderson OS, Nahar MS, Jones TR, Sartor MA, Dolinoy DC (2016). Detection of differential DNA methylation in repetitive DNA of mice and humans perinatally exposed to bisphenol a. Epigenetics.

[19] Herberth G, Herzog T, Hinz D, Roder S, Schilde M, Sack U, Diez U, Borte M, Lehmann I (2013). Renovation activities during pregnancy induce a Th2 shift in fetal but not in maternal immune system. Int J Hyg Environ Health.

[20] Weisse K, Winkler S, Hirche F, Herberth G, Hinz D, Bauer M, Roder S, Rolle-Kampczyk U, von Bergen M, Olek S (2013). Maternal and newborn vitamin D status and its impact on food allergy development in the German LINA cohort study. Allergy.

[21] Hinz D, Simon JC, Maier-Simon C, Milkova L, Roder S, Sack U, Borte M, Lehmann I, Herberth G (2010). Reduced maternal regulatory T cell numbers and increased T helper type 2 cytokine production are associated with elevated levels of immunoglobulin E in cord blood. Clin Exp Allergy.

[22] de Onis M, Martorell R, Garza C, Lartey A, Reference WMG (2006). WHO child growth standards based on length/height, weight and age. Acta Paediatr.

[23] Feltens R, Roeder S, Otto W, Borte M, Lehmann I (2015). Evaluation of population and individual variances of urinary phthalate metabolites in terms of epidemiological studies. J Chromatogr Sep Tech.

[24] Remane D, Grunwald S, Hoeke H, Mueller A, Roeder S, von Bergen M, Wissenbach DK (2015). Validation of a multi-analyte HPLC-DAD method for determination of uric acid, creatinine, homovanillic acid, niacinamide, hippuric acid, indole-3-acetic acid and 2-methylhippuric acid in human urine. J Chromatogr B.

[25] Aryee MJ, Jaffe AE, Corrada-Bravo H, Ladd-Acosta C, Feinberg AP, Hansen KD, Irizarry RA (2014). Minfi: a flexible and comprehensive bioconductor package for the analysis of Infinium DNA methylation microarrays. Bioinformatics.

[26] Du P, Zhang X, Huang CC, Jafari N, Kibbe WA, Hou L, Lin SM (2010). Comparison of beta-value and M-value methods for quantifying methylation levels by microarray analysis. BMC Bioinf.

[27] Gervin K, Page CM, Aass HC, Jansen MA, Fjeldstad HE, Andreassen BK, Duijts L, van Meurs JB, van Zelm MC, Jaddoe VW (2016). Cell type specific DNA methylation in cord blood: a 450K-reference data set and cell count-based validation of estimated cell type composition. Epigenetics.

[28] Gervin K, Hansen KD. FlowSorted.CordBloodNorway.450k: Illumina HumanMethylation data on sorted cord blood cell populations. R package version 1.4.0. 2017. https://bitbucket.com/kasperdanielhansen/Illumina_CordBlood.

[29] Junge KM, Bauer T, Geissler S, Hirche F, Thurmann L, Bauer M, Trump S, Bieg M, Weichenhan D, Gu L (2016). Increased vitamin D levels at birth and in early infancy increase offspring allergy risk-evidence for involvement of epigenetic mechanisms. J Allergy Clin Immunol.

[30] Bauer M, Fink B, Thurmann L, Eszlinger M, Herberth G, Lehmann I (2016). Tobacco smoking differently influences cell types of the innate and adaptive immune system-indications from CpG site methylation. Clin Epigenetics.

[31] Bauer T, Trump S, Ishaque N, Thurmann L, Gu L, Bauer M, Bieg M, Gu Z, Weichenhan D, Mallm JP (2016). Environment-induced epigenetic reprogramming in genomic regulatory elements in smoking mothers and their children. Mol Syst Biol.

[32] Trump S, Bieg M, Gu Z, Thürmann L, Bauer T, Bauer M, Ishaque N, Röder S, Gu L, Herberth G, Lawerenz C, Borte M, Schlesner M, Plass C, Diessl N, Eszlinger M, Mücke O, Elvers HD, Wissenbach DK, von Bergen M, Herrmann C, Weichenhan D, Wright RJ, Lehmann I, Eils R (2016). Prenatal maternal stress and wheeze in children: novel insights into epigenetic regulation. Sci Rep..

[33] Du P, Zhang XA, Huang CC, Jafari N, Kibbe WA, Hou LF, Lin SM. Comparison of Beta-value and M-value methods for quantifying methylation levels by microarray analysis. BMC Bioinf. 2010;11. 10.1186/1471-2105-11-587 PMC3012676 21118553

[34] Hayes AF (2013). Introduction to mediation, moderation, and conditional process analysis: a regression-based approach.

[35] Rodriguez-Gabin AG, Almazan G, Larocca JN (2004). Vesicle transport in oligodendrocytes: probable role of Rab40c protein. J Neurosci Res.

[36] Li Y, Jia Q, Wang Y, Li F, Jia Z, Wan Y (2015). Rab40b upregulation correlates with the prognosis of gastric cancer by promoting migration, invasion, and metastasis. Med Oncol (Northwood, London England).

[37] Takahashi M, Kamei Y, Ezaki O (2005). Mest/Peg1 imprinted gene enlarges adipocytes and is a marker of adipocyte size. Am J Physiol Endocrinol Metab.

[38] Kamei Y, Suganami T, Kohda T, Ishino F, Yasuda K, Miura S, Ezaki O, Ogawa Y (2007). Peg1/Mest in obese adipose tissue is expressed from the paternal allele in an isoform-specific manner. FEBS Lett.

[39] Soubry A, Murphy SK, Wang F, Huang Z, Vidal AC, Fuemmeler BF, Kurtzberg J, Murtha A, Jirtle RL, Schildkraut JM (2015). Newborns of obese parents have altered DNA methylation patterns at imprinted genes. Int J Obes.

[40] Karbiener M, Glantschnig C, Pisani DF, Laurencikiene J, Dahlman I, Herzig S, Amri EZ, Scheideler M (2015). Mesoderm-specific transcript (MEST) is a negative regulator of human adipocyte differentiation. Int J Obes (Lond).

[41] Kadeta Y, Kawakami T, Suzuki S, Sato M. Involvment of Mesoderm-specific Transcript in Cell Growth of 3T3-L1 Preadipocytes. J Health Sci. 2009;55(5):814-9.

[42] Nikonova L, Koza RA, Mendoza T, Chao PM, Curley JP, Kozak LP (2008). Mesoderm-specific transcript is associated with fat mass expansion in response to a positive energy balance. FASEB J.

[43] Koza RA, Nikonova L, Hogan J, Rim JS, Mendoza T, Faulk C, Skaf J, Kozak LP (2006). Changes in gene expression foreshadow diet-induced obesity in genetically identical mice. PLoS Genet.

[44] Voigt A, Agnew K, van Schothorst EM, Keijer J, Klaus S (2013). Short-term, high fat feeding-induced changes in white adipose tissue gene expression are highly predictive for long-term changes. Mol Nutr Food Res.

[45] Jura M, Jaroslawska J, Chu DT, Kozak LP (2016). Mest and Sfrp5 are biomarkers for healthy adipose tissue. Biochimie..

[46] Trapphoff T, Heiligentag M, El Hajj N, Haaf T, Eichenlaub-Ritter U (2013). Chronic exposure to a low concentration of bisphenol a during follicle culture affects the epigenetic status of germinal vesicles and metaphase II oocytes. Fertil Steril.

[47] Kaneda M, Okano M, Hata K, Sado T, Tsujimoto N, Li E, Sasaki H (2004). Essential role for de novo DNA methyltransferase Dnmt3a in paternal and maternal imprinting. Nature.

[48] El Hajj N, Pliushch G, Schneider E, Dittrich M, Muller T, Korenkov M, Aretz M, Zechner U, Lehnen H, Haaf T (2013). Metabolic programming of MEST DNA methylation by intrauterine exposure to gestational diabetes mellitus. Diabetes.

[49] Carless MA, Kulkarni H, Kos MZ, Charlesworth J, Peralta JM, Goring HH, Curran JE, Almasy L, Dyer TD, Comuzzie AG (2013). Genetic effects on DNA methylation and its potential relevance for obesity in Mexican Americans. PLoS One.

[50] Teeguarden J, Hanson-Drury S, Fisher JW, Doerge DR (2013). Are typical human serum BPA concentrations measurable and sufficient to be estrogenic in the general population?. Food Chem Toxicol.

[51] Teeguarden JG, Hanson-Drury S (2013). A systematic review of bisphenol a “low dose” studies in the context of human exposure: a case for establishing standards for reporting "low-dose" effects of chemicals. Food Chem Toxicol.

