# Supplementary material for: MEST mediates the impact of prenatal bisphenol A exposure on long-term body weight development
Source: Clin Epigenetics. 2018 Apr 20;10:58. doi: 10.1186/s13148-018-0478-z (PMC5910578; doi:10.1186/s13148-018-0478-z)
Supplement: Supplementary file 1 — Table S1. Primer for gene expression analysis. Table S2. Mediator model for the association of prenatal BPA exposure with cord blood MEST DNA methylation and expression (according to Fig. 1c). Table S3. Mediator model for the association of prenatal BPA exposure with cord blood MEST DNA methylation and children’s BMI z scores at year 1 (according to Fig. 3a). Table S4. Mediator model for the association of cord blood MEST DNA methylation and children’s BMI z scores at years 1 and 6 (according to Fig. 3b). (DOCX 45 kb) [file 13148_2018_478_MOESM1_ESM.docx]

**Supplementary Material**

***MEST* mediates the impact of prenatal bisphenol A exposure on long-term body weight development**

Kristin M Junge^a*^  & Beate Leppert^a*^, Susanne Jahreis^a,b^, Dirk K. Wissenbach^c,d^, Ralph Feltens^c^, Konrad Grützmann^a,e^, Loreen Thürmann^a,r^, Tobias Bauer^f^, Naveed Ishaque^f^, Matthias Schick^g^, Melanie Bewerunge-Hudler^g^, Stefan Röder^a^, Mario Bauer^a^, Angela Schulz^h,^,Michael Borte^i^, Kathrin Landgraf^Ij,k^, Antje Körner^j,k^ Wieland Kiess^j,k^, Martin von Bergen^c,l^, Gabriele I. Stangl^m,n^, Saskia Trump^a^, Roland Eils^o,p,q*^ & Tobias Polte^a,b*^ & Irina Lehmann^a,r*^

a Helmholtz Centre for Environmental Research (UFZ), Department of Environmental Immunology, Leipzig, Germany

b Leipzig University Medical Center, Department of Dermatology, Venerology and Allergology, Leipzig, Germany

c Helmholtz Centre for Environmental Research (UFZ), Department Molecular Systems Biology, Leipzig, Germany

d University Hospital Jena, Institute of Forensic Medicine, Jena, Germany

e Core Unit for Molecular Tumor Diagnostics (CMTD), National Center for Tumor Diseases (NCT) Dresden, D-01307 Dresden, Germany; German Cancer Consortium (DKTK), Dresden, Germany; German Cancer Research Center (DKFZ), 69120 Heidelberg, Germany

f German Cancer Research Center (DKFZ), Division of Theoretical Bioinformatics, Heidelberg, Germany

g German Cancer Research Center (DKFZ), Genomics and Proteomics Core Facility, Heidelberg, Germany

h University of Leipzig, Medical Faculty, Rudolf-Schönheimer-Institute of Biochemistry, Leipzig, Germany

i Children’s Hospital, Municipal Hospital “St. Georg”, Leipzig, Germany

j University of Leipzig, LIFE - Leipzig Research Centre for Civilization Diseases, Leipzig, Germany.

k University of Leipzig, Hospital for Children and Adolescents - Centre for Pediatric Research, Leipzig, Germany.

l University of Leipzig, Faculty of Biosciences, Pharmacy and Psychology, Institute of Biochemistry, Leipzig, Germany

m Martin Luther University Halle-Wittenberg, Institute of Agriculture and Nutritional Sciences, Halle (Saale), Germany

n Competence Cluster for Nutrition and Cardiovascular Health (nutriCARD) Halle-Jena Leipzig, Germany

o German Cancer Research Center (DKFZ), Heidelberg Center for Personalized Oncology, DKFZ-HIPO, Heidelberg, Germany

p Berlin Institute of Health and Charité, Center for Digital Health, Berlin

q Heidelberg University Hospital, Health Data Science Unit, Heidelberg

r Berlin Institute of Health, Unit for Molecular Epidemiology, Berlin

* authors contributed equally

**Detailed methods description**

***Analyses of urinary bisphenol A concentration in human samples***

BPA quantification was carried out for 552 maternal urine samples (34^th^ week of gestation) using a multianalyte procedure as described by Feltens *et al.*.

For this, 100 µl aliquots were buffered with 150 mM Na·ADA (N-(2-acetamido)-iminodiacetic acid (ADA), from Fluka (Taufkirchen, Germany)), pH 6.6, spiked with 25 µl 50 % methanol (LC-MS grade from Merck (Darmstadt, Germany)) containing 20 ng of ^13^C_12_-labelled BPA, 2 ng of ^13^C_4_ methylumbelliferone (LGC (Wesel, Germany)) and unlabeled methylumbelliferone-glucuronide (Sigma-Aldrich (Seelze, Germany)), and incubated for 5 h at 37 °C with 3.5 µl deglucuronidase / arylsulfatase (Roche (Mannheim, Germany)) in a total volume of 210 µl to achieve deglucuronation of the metabolites. Reactions were stopped by addition of 510 µl 700 mM formic acid (FA) with 47 mM acetic acid (Biosolve (Valkenswaard, The Netherlands)). Purification of samples was performed via solid phase extraction (SPE) on Isolute C18 columns (Biotage (Düsseldorf, Germany)) via gravity flow. After equilibration of the columns using 3 ml 100 % methanol, followed by 3 ml of 2 % FA in 5 % methanol, samples were applied, followed by two washing steps of 3 ml 2 % FA in 10 % methanol. Analytes were eluted in 1.5 ml of 2 % FA in 90 % methanol, vacuum-dried at 45 °C, resuspended in 100 µl 35 % acetonitrile (LGC Promochem (Wesel, Gemany)) and transferred to insert-containing autosampler vials, which were crimp-sealed.

10 µl aliquots of these extracts were analyzed on a Dionex™ UltiMate™ 3000 UPLC System (Thermo Scientific, MA, USA) using a sigmoidal 21 min gradient from 5 % to 100 % methanol (eluent B) with 0.01 % FA.

Separation was achieved via reversed phase chromatography on an Acquity UPLC BEH C18, 1.7 µm, 2.1 mm x 100 mm column (Waters, Eschborn, Germany) at 50 °C and 200 µl/min. Detection and quantification of analytes after elution and post-column infusion (50 µl/min in 0.2 % NH_3_) was achieved on a Q-Trap 5500 triple quadrupole mass spectrometer (AB Sciex, Framingham, MA, USA) with electrospray ionisation at 350 °C and 4500 V in negative mode via previously established, scheduled SRMs. For BPA *m/z* = 227 to 212 and 227 to 139 da , for ^13^C_12_ BPA *m/z* = 239 to 224 and 239 to 139 da was used. Between samples, the column was washed for 5.5 min with 100 % methanol, 0.01 % FA and an injection of 100 µl isopropanol/acetonitrile/acetone (45:45:10), followed by re-equilibration in 5 % methanol, 0.01 % FA. Absolute concentrations of BPA and the deglucuronation standard MeUmb were calculated with respect to the known, spiked-in concentrations of the isotopically labelled standards and previously obtained calibration curves, using dedicated software (Analyst, AB Sciex). Finally, concentrations were normalized against urinary creatinine concentrations. All samples were analyzed once with the described LC-MS/MS procedure.

***In vitro adipocytes model***

Human adipose-derived mesenchymal stem cells (MSC) were purchased from ATCC® (PCS-500-011; #59753760), seeded in cell culture flasks at 5000 cells/cm^2^ in MSC Basal Medium (BM; ATCC®, PCS-500-030; supplemented with 0.1 % Phenol Red, 3.2 % MSC Growth Kit (ATCC®, PCS-500-040), 0.2 % Gentamicin /Amphotericin B and 0.1 % Penicillin/Streptomycin) and maintained at 37 °C and 5 % CO_2_. Medium was changed every other day until MSCs reached 70 % confluence. For adipocyte differentiation MSCs seeded at 2880 cells/well in a 96 well microelectrode plate from ACEA Biosciences Inc. (E-Plate 96 View PET, gold electrodes, 400 µm window for microscopy, well area 0.2 cm^2^).

For adipocyte differentiation cells were fed with Adipocyte Differentiation Initiation Medium (ADIM; ATCC® Adipocyte Differentiation Toolkit PCS-500-050; Adipocyte Basal Medium supplemented with 7 % AD Supplement). ADIM was exchanged after 48 h and after another 48 h changed to Adipocyte Maintenance Medium (ADMM; ATCC®, Adipocyte Differentiation Toolkit PCS-500-050, Adipocyte Basal Medium supplemented with 5.5 % ADM Supplement). Further on, medium was exchanged every 48 h to 96 h according to the manufacturer’s instructions. Adipocyte differentiation ended after a total of 13 days with ADMM.

Cells were treated with a 10 or 50 µM BPA (working solution in 0.05 % Ethanol) during the whole differentiation period; freshly added after every medium change. The differentiation process was monitored in real-time with the impedance based xCELLigence SP System from Roche® on a microelectrode 96 well E-Plate. The plate was placed into the SP working station in a humidified incubator at 37 °C and 5 % CO2 and a background (blank) measurement was taken. The growth rate was monitored every 10 min by electrical impedance measurements that were paused for media changes.

After the differentiation, cells were stained with Oil Red O for triglyceride depots and mRNA analysis was performed. For Oil Red staining, differentiated adipocytes were fixed with 10 % formaldehyde for 1 min at RT, washed 3 times with PBS and stained with a filtered Oil Red O solution (0.3 % (w/v)) for 45 min at RT. Afterwards, wells were washed 3 times with aqua dest. and viewed at 20x magnification under bright field conditions with an inverted microscope.

For mRNA analysis, differentiated adipocytes were harvested in PeqGold and stored at -80 °C for at least 24 h. Total mRNA isolation was performed with PeqGold Phase Trap tubes and RNA precipitation was done according to manufacturer’s instructions. qPCR was performed with a self-made SYBR Green based master mix in a Roche®LightCycler480. Gene expression of *peroxisome proliferator-activated receptor gamma* (*PPARG),* sterol regulatory element-binding factor 1(*SREBF1), lipoprotein lipase (LPL), leptin (LEP), fatty acid synthase (FASN), mesoderm specific transcript (MEST*), *estrogen receptor alpha* (*ESR1)* and *insulin receptor substrate 2* (*IRS2)* were normalized to *Glycerinaldehyd-3-phosphat-Dehydrogenase (GAPDH)* expression.

To test for cytotoxic effects of BPA, a MTT (3-(4,5-Dimethylthiazol-2-yl)-2,5-diphenyltetrazoliumbromid) assay was performed. Therefore MSCs were seeded in triplicates in a 96 well plate, settled for 48 h, and treated with BPA for another 48 h. 10 µM MTT solution was applied and incubated for 4 h at 37 °C. Afterwards 100 µl stop-solution was added and incubated over night at 37 °C. Extinction was measured at 570 nm using a Synergy Microplate Reader from BioTek and Gen5^TM^ Software.

***DNA methylation analysis***

***Illumina methylation array.***

For 472 children a genome-wide screen of DNA methylation pattern was performed by using the Infinium HumanMethylation450 BeadChip (Illumina, San Diego, US) arrays, allowing the simultaneous quantitative measurement of the methylation status at 485,577 CpG sites. The DNA quality was checked by agarose-gel analysis, and samples with an average fragment size >3kb were selected for methylation analysis. DNA concentrations were determined using PicoGreen (Life Technologies, Darmstadt, Germany). Genomic DNA (500 ng) from each sample was bisulfite converted using the EZ-96 DNA Methylation Kit (Zymo Research Corporation, Orange, US) according to the manufacturer recommendations. After bisulfite conversion, each sample was whole genome amplified and enzymatically fragmented following the instructions in the Illumina Infinium HD Assay Methylation Protocol Guide. About 200 ng DNA was applied to Infinium HumanMethylation450 BeadChip and hybridization is performed for 16-24h at 48°C.

Allele-specific primer annealing is followed by single-base extension using DNP- and Biotin-labeled ddNTPs. After extension, the array is fluorescently stained, scanned, and the intensities of the non-methylated and methylated bead types were measured. Microarray scanning was done using an iScan array scanner. Data was normalized using the SWAN (subset-quantile within array normalization) method of the minfi R package [1]. DNA methylation values, described as beta values (β), are recorded for each locus in each sample. Beta values represent the ratio of the intensity of the methylated bead type to the combined locus intensity. For statistical analyses β‑values were transferred to M-values [2]. Probes were filtered by annotation of the minfi (SNPs) and RnBeads (cross-reactivity) packages for R (Bioconductor version 3.5). Only probes with a detection p-values <0.05 in all samples were retained for analyses (n=480,021). Further, we successively filtered out probes detecting SNPs or CH loci (n= 3,050), probes overlapping SNPs at the interrogation site or at the single nucleotide extension (n=17,343), and cross-reactive probes (n=26,237). Finally, only probes mapping to autosomes were considered leaving a total of 422,157 CpGs for down-stream analysis.

***Gene expression analysis in the LINA cohort***

Gene expression was performed as reported [3] by 96.96 Dynamic Array (Fluidigm, San Francisco, CA, USA). Briefly, intron-spanning primers were designed and UPL probes were selected by the Universal Probe Library Assay Design Center (http://qpcr.probefinder.com/organism.jsp.). A preamplification reaction was performed by pooling all primers (final concentration, 50 nM), 5 µl of cDNA and 2x PreAmp Master Mix (Applied Biosystems/Life Technologies GmbH, Darmstadt, Germany). The cycling program consisted of 95°C for 10 min, followed by 14 cycles of 95°C for 15 sec and 60°C for 4 min on a LightCycler 480 (Roche Applied Science, Mannheim, Germany). The qPCRs of 1:5 diluted with TE buffer preamplified templates were performed following manufacture’s instruction for UPL (Roche, Mannheim, Germany) assays. Briefly, for each individual assay, a 10X Assay Mix that contained 2 µM of each forward and reverse primers, 1 µM UPL probe and 0.025% Tween-20 was prepared, and 5 µl of the mix was loaded into the assay inlets of the array. Into the sample inlets, 5 µl of the following solution was dispensed: 2.5 µl of PreAmp sample in 1.1X of FastStart Universal Probe Master Mix (Roche, Mannheim, Germany). The cycling program consisted of 2 min at 50°C, 10 min at 95°C, followed by 35 cycles of 95°C for 15 sec, 1 min at 60°C and 70°C for 5 sec. All reactions were performed in triplicates. Gene expression values were determined by using the 2-∆∆CT method[4] and GAPD and GUSB as reference genes and normalized to the lowest measured value. Following primer were used for MEST (primer-for 5’- atcgtggaagcgcttttg, -rev 5’- gaccagatcgattctgcttgta, UPL50) and reference genes GAPD and GUSB (primer-for 5’-gctctctgctcctcctgttc, -rev 5’-acgaccaaatccgttgactc, UPL 60; -for 5’-cgccctgcctatctgtattc, -rev 5’-tccccacagggagtgtgtag, UPL 57, respectively).

***References***

1. Aryee MJ, Jaffe AE, Corrada-Bravo H, Ladd-Acosta C, Feinberg AP, Hansen KD, Irizarry RA: **Minfi: a flexible and comprehensive Bioconductor package for the analysis of Infinium DNA methylation microarrays**. *Bioinformatics* 2014, **30**(10):1363-1369.

2. Du P, Zhang X, Huang CC, Jafari N, Kibbe WA, Hou L, Lin SM: **Comparison of Beta-value and M-value methods for quantifying methylation levels by microarray analysis**. *BMC Bioinformatics* 2010, **11**:587.

3. Bauer M, Grabsch C, Schlink U, Klopp N, Illig T, Kramer U, von Berg A, Schaaf B, Borte M, Heinrich J *et al*: **Genetic association between obstructive bronchitis and enzymes of oxidative stress**. *Metabolism* 2012, **61**(12):1771-1779.

4. Livak KJ, Schmittgen TD: **Analysis of relative gene expression data using real-time quantitative PCR and the 2(-Delta Delta C(T)) Method**. *Methods* 2001, **25**(4):402-408.

5. Bauer M, Grabsch C, Schlink U, Klopp N, Illig T, Kramer U, von Berg A, Schaaf B, Borte M., Heinrich J, Herbarth O, Lehmann I, and Roder S: **Genetic association between obstructive bronchitis and enzymes of oxidative stress.** *Metabolism* 2012 **61**, 1771-1779

6. Livak KJ, and Schmittgen TD: **Analysis of relative gene expression data using real-time quantitative PCR and the 2(-Delta Delta C(T))** Method. *Methods* (2001) **25**, 402-408

**Supplementary Tables**

Table S1. **Primer for gene expression analysis**

| Gen | forward 5’→3’ | reverse 5’→3’ |
| --- | --- | --- |
| human qPCR primer | | |
| *GAPDH* | CTCTCTGCTCCTCCTTTCGAC | TGAGCGATGTGGCTCGGCT |
| *PPARγ* | TTACGCCTCGGTGTTTAGGG | TGGTCATTTCGTTAAAGGCTGA |
| *LEP* | TTTCACACACGCAGTCAGTC | GTGGAGCCCAGGAATGAAGT |
| *LPL* | CCGCCGACCAAAGAAGAGAT | TAGCCACGGACTCTGCTACT |
| *hCEBPα* | GGAGCAAATCGTGCCTTGTC | TTCTCTCATGGGGGTCTGCT |
| *FASN* | GTCTTGAACTCCTTGGCGGA | AGGAAGATAGCCATGCCGAG |
| *IRS2* | GTGAAAGAGTGAAGATCTGTCTGG | TTGCCTTGTTGGTGCCTCAT |
| *SREBF1* | GCTCCCTAGGAAGGGCCGTA | AAGTGCAATCCATGGCTCCG |
| *ESR1* | TGGCCCAGCTCCTCCTCATCCTC | AGTGGCTTTGGTCCGTCTCCTC |
| *MEST* | ATCGGGTGATTGCCCTTGATT | GAAAGAAGGTTGATCCTGCGG |
| murine qPCR primer |  |  |
| *Gapdh* | CCTGCTTCACCACCTTCTTGA | TGTGTCCGTCGTGGATCTGA |
| *Mest* | AGAGTGGTGGGTCCAAGTAGG | AAGCACAACTATCTCAGGGCT |

Table S2. **Mediator model for the association of prenatal BPA exposure with cord blood *MEST* DNA methylation and expression** (according to Figure 1C)**.**

| **parameter** | **effect size** | **95% CI** | ***p*-value** |
| --- | --- | --- | --- |
| a | **-0.04** | **-0.63, -0.48** | **0.011** |
| b | **-11.90** | **-21.34, -2.45** | **0.014** |
| c (direct effect) | -0.02 | -0.41, 0.37 | 0.911 |
| ab (indirect effect) | **0.47** | **0.07, 1.24** | **<0.05*** |

* significant *p*-value derived according to 95% CI range

Table S3. **Mediator model for the association of prenatal BPA exposure with cord blood *MEST* DNA methylation and children’s BMI z-scores at year 1** (according to Figure 3A)**.**

| **parameter** | **effect size** | **95% CI** | ***p*-value** |
| --- | --- | --- | --- |
| a | **-0.49** | **-0.62, -0.36** | **<0.001** |
| b | **-4.22** | **-8.18, -0.26** | **0.037** |
| c (direct effect) | -0.01 | -0.19, 0.17 | 0.934 |
| ab (indirect effect) | **0.29** | **0.03, 1.09** | **<0.05*** |

* significant *p*-value derived according to 95% CI range

Table S4. **Mediator model for the association of cord blood *MEST* DNA methylation and children’s BMI z-scores at year 1 and year 6** (according to Figure 3B)**.**

| **parameter** | **effect size** | **95% CI** | ***p*-value** |
| --- | --- | --- | --- |
| a | -0.34 | -0.75, 0.08 | 0.113 |
| b | **0.53** | **0.40, 0.67** | **<0.001** |
| c (direct effect) | -0.07 | -0.46, 0.32 | 0.716 |
| ab (indirect effect) | **-0.18** | **-0.51, -0.06** | **<0.05*** |

* significant *p*-value derived according to 95% CI range

**Supplementary Figure Legends**

**Figure S1:** Shown are the location of the *MEST* gene on chromosome 7 (upper part), the 450K array CpG in the *MEST* promoter (middle part) and the region covered by the MassARRAY amplicon within the promoter region (CpG sites are depicted in red).

**Figure S2:** **BPA effect on weight development assessed in a murine in vivo model stratified for gender.** Shown are means and standard deviation from n≥8 mice/group for all, female and male mice separately. *p*-values are derived from ANOVA.

**Figure S3. MTT assay:** MTT test for cell viability after exposure to BPA and the solvent control EtOH (0.05%), normalized to unexposed control, Student’s t-test *p<0.05, mean±SD, n=3

**Figure S4.** **Summary scheme:** Results overview and hypothesis indicating the influence of prenatal BPA exposure on *MEST* methylation and expression that is associated with adipocyte differentiation and overweight development in infant offspring.
