# Supplementary material for: MEST mediates the impact of prenatal bisphenol A exposure on long-term body weight development
Source: Clin Epigenetics. 2018 Apr 20;10:58. doi: 10.1186/s13148-018-0478-z (PMC5910578; doi:10.1186/s13148-018-0478-z)
Supplement: Supplementary file 2 — Figure S1. Shown are the location of the MEST gene on chromosome 7 (upper part), the 450 K array CpG in the MEST promoter (middle part) and the region covered by the MassARRAY amplicon within the promoter region (CpG sites are depicted in red). (PDF 32 kb) [file 13148_2018_478_MOESM2_ESM.pdf]

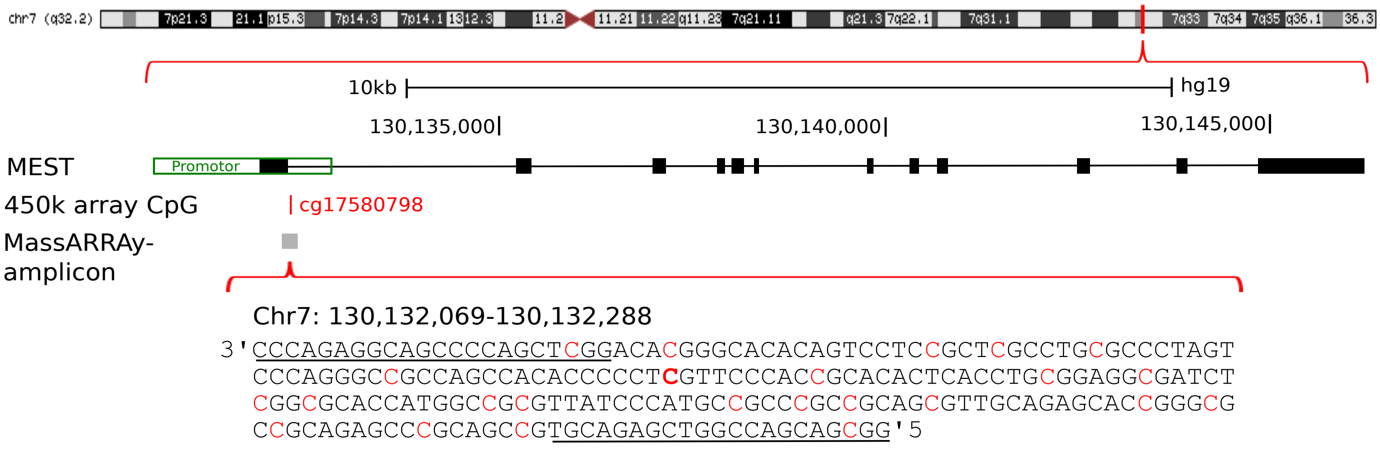
chr7 (q32.2)
10kb
hg19
130,135,000
130,140,000
130,145,000
MEST
Promotor
450k array CpG
cg17580798
MassARRAy-amplicon
Chr7: 130,132,069-130,132,288
3'CCCAGAGGCAGCCCCAGCTCGGACACGGGCACACAGTCCTCCGCTCGCCTGCGCCCTAGT
CCCAGGGCCGCCAGCCACACCCCCTCGTTCCCACCGCACACTCACCTGCGGAGGCGATCT
CGGCGCACCATGGCCGCGTTATCCCATGCCGCCCGCCGCAGCGTTGCAGAGCACCGGGCG
CCGCAGAGCCCGCAGCCGTGCAGAGCTGGCCAGCAGCGG'5
