# Supplementary figures and images for: MEST mediates the impact of prenatal bisphenol A exposure on long-term body weight development
Source: Clin Epigenetics. 2018 Apr 20;10:58. doi: 10.1186/s13148-018-0478-z (PMC5910578; doi:10.1186/s13148-018-0478-z)

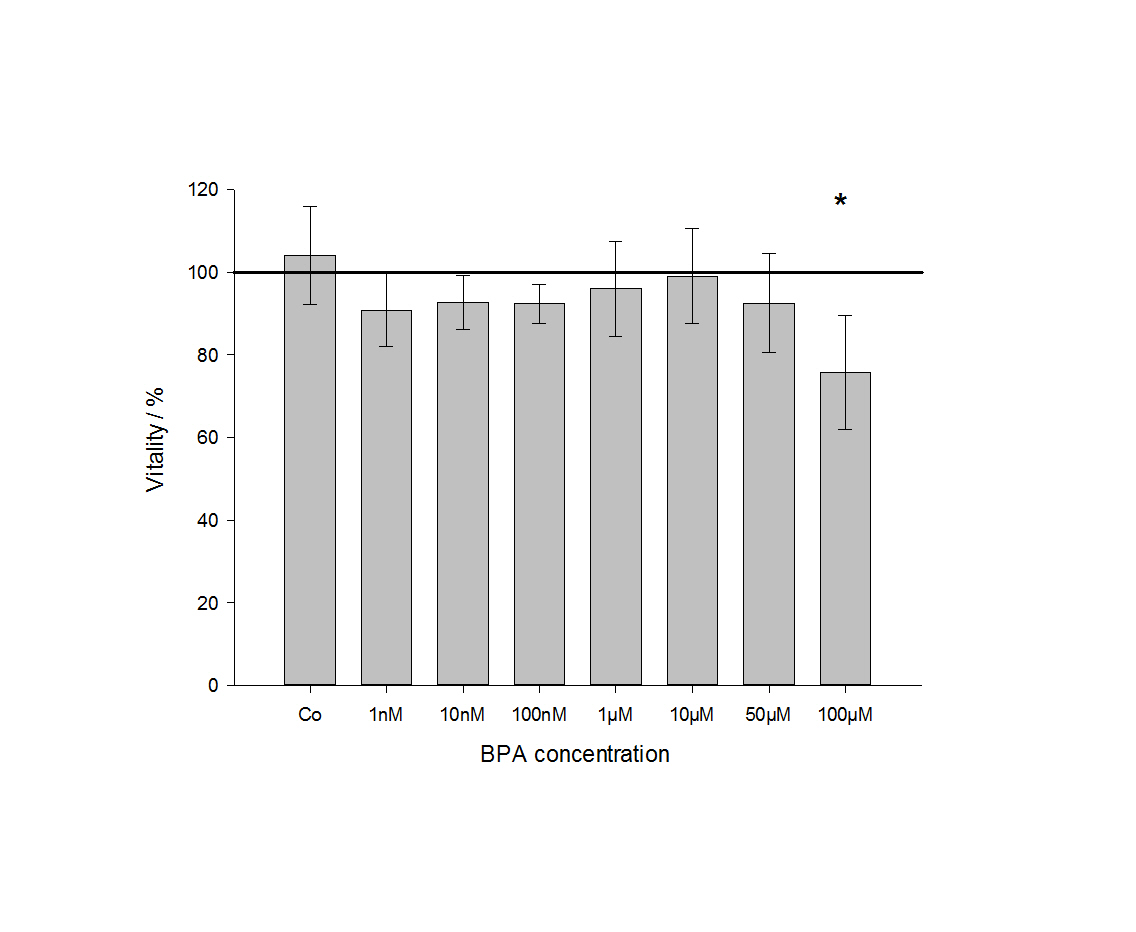

Supplement: Supplementary file 4 — Figure S3. MTT assay: MTT test for cell viability after exposure to BPA and the solvent control EtOH (0.05%), normalized to unexposed control, Student’s t test *p < 0.05, mean ± SD, n = 3. (JPEG 105 kb) [file 13148_2018_478_MOESM4_ESM.jpg]

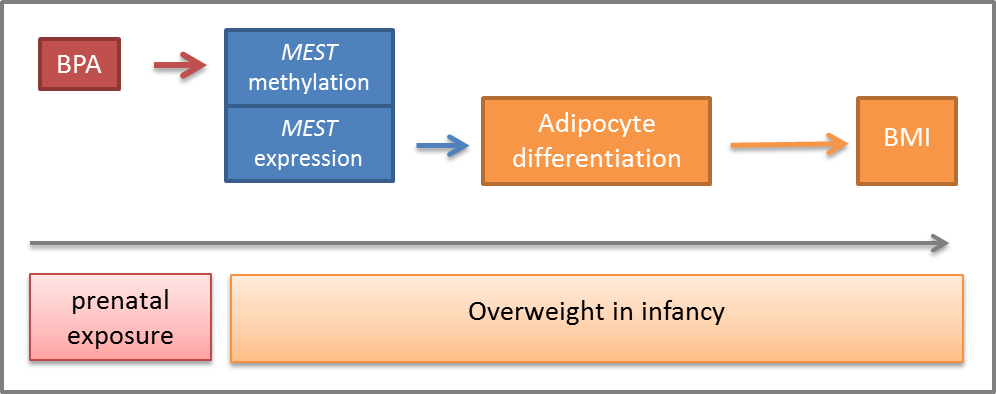

Supplement: Supplementary file 5 — Figure S4. Summary scheme: results overview and hypothesis indicating the influence of prenatal BPA exposure on MEST methylation and expression that is associated with adipocyte differentiation and overweight development in infant offspring. (PNG 19 kb) [file 13148_2018_478_MOESM5_ESM.png]
